# Editorial: Cognitive Empathy and Perspective Taking: Understanding the Mechanisms of Normal and Abnormal Experiences and Abilities

**DOI:** 10.3389/fpsyt.2022.945258

**Published:** 2022-06-02

**Authors:** Renate L. E. P. Reniers, Ahmad Abu-Akel, Ana Seara-Cardoso

**Affiliations:** ^1^Institute of Clinical Sciences, University of Birmingham, Birmingham, United Kingdom; ^2^Institute for Mental Health, University of Birmingham, Birmingham, United Kingdom; ^3^Institute of Psychology, University of Lausanne, Lausanne, Switzerland; ^4^School of Psychological Sciences, University of Haifa, Haifa, Israel; ^5^Centro de Investigaçāo em Psicologia, Escola de Psicologia, Universidade do Minho, Braga, Portugal

**Keywords:** cognitive empathy, perspective taking, neural mechanisms, behavioral mechanisms, clinical

Human behavior is largely based on our understanding and interpretation of the feelings and actions of others. In order to function in and adapt to this social world, we rely on social cognitive processes such as empathy and perspective taking (1, 2). Empathy is now commonly characterized as consisting of cognitive and affective components. Cognitive empathy is defined as the ability to construct a working model of the emotional states of others and importantly entails the comprehension of another person's emotional experience. This can be achieved by actively imagining what another person may be feeling or by intuitively putting oneself in another person's position; processes joined under the header perspective taking (2). This Research Topic aims to provide a more comprehensive picture of the mechanisms underlying cognitive empathy and perspective taking. By collating research consisting of neuroimaging discoveries, together with detailed neuropsychological and behavioral findings in healthy, clinical, and at-risk populations, we aim to increase understanding of the neural and behavioral mechanisms of normal and abnormal cognitive empathic experiences and perspective taking abilities.

Our ability to understand another person's internal states relies on the integration of our representations of this person's feelings with our beliefs about their feelings within specific contexts (2, 3). One such specific context is that of Thought Action Fusion (TAF), a form of magical thinking where internal thoughts are perceived to exert equivalent effects to external actions. Eddy and Hansen showed that emotional, but not cognitive, aspects of empathy were associated with TAF and that alexithymia partially mediated these associations. In the specific context of empathy for pain, Zebarjadi et al. demonstrated that neural oscillatory modulations and their cortical sources presented patterns corresponding to multiple facets of empathy, thereby providing further empirical support for a more graded neurophenomenological framework of empathy.

While integrating our representations of another person's feelings with our beliefs about their feelings, we maintain the distinction between our own and other's internal states (4). Within this context, Ribeiro da Costa et al. investigated the interplay between the default mode network (DMN) and salience network (SN). Anterior and posterior DMN regions exhibited increased functional connectivity during social task performance compared to resting state. Watching emotional videos of their romantic partner and elaborating on their partner's experience revealed more limited SN's connectivity in participants in comparison to elaboration on their own experience and the Rest condition. These findings highlight an interplay between the DMN and SN networks in the context of *self* vs. *other* experiences.

Considering that an empathic interaction may last beyond the initial response, Arbel et al. used a novel task to demonstrate an association between adaptive empathy, conceptualized as the ability to learn and adjust one's empathic responses based on feedback, and trait cognitive empathy. Their results underscore the role of learning in influencing the dynamics and outcomes of social interactions, but which may be susceptible to inter-individual differences in mentalizing abilities.

Deficits in cognitive empathy and perspective taking are well-documented in clinical populations such as individuals with schizophrenia spectrum disorders (SSD), autism spectrum disorders (ASD), and antisocial behavior (5–7). In their literature review, Chang et al. suggest that any dysfunction in cognitive empathy associated with antisociality varies by subtype of the antisocial individual and is specific to subcomponents of cognitive empathy. Individuals of the psychopathic subtype fail to implicitly engage in cognitive empathy, and potentially lack insight into this issue, but show an ability to engage in cognitive empathy when explicitly required. Individuals of the antisocial-only subtype appear able to engage in cognitive empathy, but may display subtle difficulties in accurately inferring the other's emotions.

Kuis et al. presented evidence for impairments in cognitive empathy in individuals in the Ultra High Risk (UHR) phase of psychosis. Self-reported levels of cognitive empathy in this group were comparable to those reported by patients with SSD, but lower than those reported by individuals without reported mental illness. More specifically, perspective-taking in this group was negatively associated with time spent on structured social activities. These findings may suggest that difficulties in interpreting the thoughts and feelings of others precede the onset of psychotic disorders. Consistent with these findings, Karpouzian-Rogers et al. demonstrated that individuals with SSD performed more poorly on a cognitive empathy task and presented with a thinner temporo-parietal junction (TPJ) than control participants. Furthermore, amongst individuals with SSD, but not amongst controls, better performance on the cognitive empathy task predicted lesser thinning of the right TPJ 2 years later. These findings suggest a predictive role of cognitive empathy ability of TPJ integrity in SSD.

Cognitive empathy deficits have also been observed in a younger sample of adolescents with ASD and adolescents with behavioral problems. Vilas et al. demonstrated that while task results were inconclusive in regards to differences in empathic accuracy between these clinical groups and typically developing adolescents, the ASD group showed lower scores in self-reported perspective taking abilities, and adolescents with behavioral difficulties reported more difficulties in imagining another person's feelings. These results not only agree with the notion that empathy deficits are present in both ASD and behavioral disorders but also underline that these deficits might be qualitatively different.

Finally, the work by Nahal et al. showed enhanced cognitive empathy in female undergraduate students, specifically in detecting negative and positive mental states. Their findings suggest that cognitive empathy is underdeveloped (with a male bias) with increased autistic traits and overdeveloped (with a female bias) with increased schizotypal traits, and highlight the centrality of imagination and focused attention in cognitive empathy.

The work presented in this Research Topic emphasizes the complexity of the empathy construct and in particular the need to dissect cognitive empathy when considering its underlying neural and behavioral mechanisms. Deficits in cognitive empathy and perspective taking abilities in individuals with SSD, ASD, and antisocial behavior are well-documented and studies presented here have highlighted qualitative differences in association with illness. Together, the studies in this Research Topic portray cognitive empathy and perspective taking as complex and dynamic experiences, underlined by abilities that are sensitive to context and disorder, and in which imagination takes a central role.

## Author Contributions

All authors listed have made a substantial, direct, and intellectual contribution to the work and approved it for publication.

## Funding

AS-C contribution was supported by the Foundation for Science and Technology (FCT) through the Portuguese State Budget (Ref.: UIDB/PSI/01662/2020).

## Conflict of Interest

The authors declare that the research was conducted in the absence of any commercial or financial relationships that could be construed as a potential conflict of interest.

## Publisher's Note

All claims expressed in this article are solely those of the authors and do not necessarily represent those of their affiliated organizations, or those of the publisher, the editors and the reviewers. Any product that may be evaluated in this article, or claim that may be made by its manufacturer, is not guaranteed or endorsed by the publisher.
